# IonFlow: a galaxy tool for the analysis of ionomics data sets

**DOI:** 10.1007/s11306-021-01841-z

**Published:** 2021-09-25

**Authors:** J. Iacovacci, W. Lin, J. L. Griffin, R. C. Glen

**Affiliations:** 1 Department of Metabolism Digestion and Reproduction, Faculty of Medicine, Imperial College London, London, UK; 2 Breast Cancer Now Toby Robins Research Centre, The Institute of Cancer Research, London, UK; 3 Department of Biochemistry and Systems Biology Centre, University of Cambridge, Cambridge, UK; 4 Department of Chemistry, Centre for Molecular Informatics, University of Cambridge, Cambridge, UK

**Keywords:** Ionomics, Network biology, Galaxy platform

## Abstract

**Introduction:**

Inductively coupled plasma mass spectrometry (ICP-MS) experiments generate complex multi-dimensional data sets that require specialist data analysis tools.

**Objective:**

Here we describe tools to facilitate analysis of the ionome composed of high-throughput elemental profiling data.

**Methods:**

IonFlow is a Galaxy tool written in R for ionomics data analysis and is freely accessible at https://github.com/wanchanglin/ionflow. It is designed as a pipeline that can process raw data to enable exploration and interpretation using multivariate statistical techniques and network-based algorithms, including principal components analysis, hierarchical clustering, relevance network extraction and analysis, and gene set enrichment analysis.

**Results and Conclusion:**

The pipeline is described and tested on two benchmark data sets of the haploid S. Cerevisiae ionome and of the human HeLa cell ionome.

**Supplementary Information:**

The online version contains supplementary material available at 10.1007/s11306-021-01841-z.

## Introduction

The multi-omics era has seen an increase in the acquisition of multivariate and megavariate datasets to describe the functional genetic patterns that arise from multiple levels of complexity of the cell, including the genome, the epigenome, the transcriptome, the metabolome, the proteome, the lipidome and the ionome (Fondi & Liò, 2015; Haas et al., 2017; Pinu et al., 2019).

In particular the ionome, defined as the elemental composition of an organism, is studied through the quantitative and simultaneous measurement of intracellular elements and changes in their composition in response to environmental and genetic perturbations (Salt et al., 2008). Inductively coupled plasma mass spectrometry (ICP-MS) is a technology used in systems biology and clinical research to profile the concentration of elements within samples and cells of living organisms (Amais et al., 2020; Barkla et al., 2016; Baxter, 2010; Konz et al., 2017; Meyer et al., 2018). This technology has been coupled with screening experiments using genetic modifications to study genome-wide genetic associations that are revealed by phenotypical cellular responses at the level of element abundances, in diverse model organisms, including yeast (Danku et al., 2009; Eide et al., 2005; Yu et al., 2012), and plants (Baxter et al., 2008; Chao et al., 2011; Salt et al., 2008).

Ionome data sets are generally analysed using multivariate statistical techniques such as principal components analysis (PCA) and clustering, as well as methods for network inference and analysis. These approaches have shown to be effective in revealing patterns of correlation between intracellular abundances of different elements and between elemental profiles of different samples (Eide et al., 2005; Iacovacci et al., 2020; Yu et al., 2012). However, at present, no bioinformatics pipeline exists that allows the performance of this type of analysis in a standardised fashion. For this reason, we developed IonFlow, an R-based Galaxy tool designed for the analysis of ionome data sets from ICP-MS experiments. The software is freely available on Galaxy (https://usegalaxy.org/, Jalili et al., 2020) and is designed to have a simple user-friendly interface.

## Materials and methods

### Workflow

**Fig. 1:**
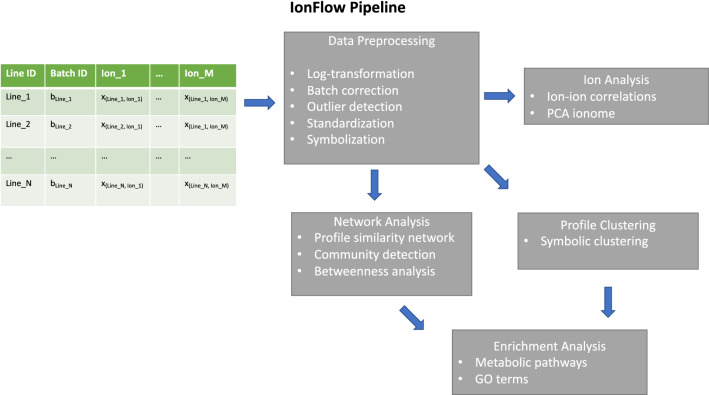
The functional architecture of the IonFlow pipeline

In Fig. 1 we schematically outline the IonFlow pipeline. The format of the raw data is a csv table describing a set of *N* measurements (rows) of the concentration of *M* distinct elements (columns). Because usually the elements are profiled via mass-spectrometry in the form of ions, we also refer to them as ‘ions’ here (but note these ions may be complex adducts). The column *Line* describes the variable associated with the *N* measurements, which can be a gene ID associated to a single-gene mutant of a model organism under study.

Multiple samples of the same line can be present (repeated line ids in the rows) and one column in the table (*Batch_ID* in figure) typically provides the batch identification number, a label that groups the measurements according to the experimental design, describing for example the samples that were measured in the same batch or that were measured in the same plate, and is used to correct the data for batch effects (Mertens, 2017).

### Data pre-processing

The *PreProcessing* function processes the raw data according to a series of sequential operations.

1. *Log-transformation* raw data are automatically log-transformed, a common practice in the analysis of mass spectrometry data, which improves analysis of features (ions concentrations) by transforming the data to a distribution that is closer to a Gaussian distribution (Mertens, 2017).
2. *Batch correction* three arguments of the function give the user a customised way to normalise the data for batch effects; these are *method_norm*, for setting the batch correction method, *control_use* which indicates how to use the control lines for batch correction, and *control_lines*, a string specifying the cell lines to be used as control. If the parameter *method_norm* is equal to “none”, no correction is performed; if “median” is assigned as a value, then all the measurements of each ion *m* in batch *b* are scaled by the median value of the corresponding ion in that batch; if “median + std” is assigned to the parameter the concentrations are further scaled by the standard deviation (std) of the ion concentrations within the batch.
  When the parameter *control_use* is set to “all” then all lines in the batch are used to compute the batch median and std; when *control_use* is set to “control” the batch median and std are computed using a subset of lines specified in the character vector *control_lines* passed to the function. Finally, if *control_use* is set to “control.out”, all lines except the control lines are used to compute the values of batch median and std.
3. *Outlier detection* after all measurements are normalised to their batches, outlier values are detected and removed according to three alternative methods that can be specified by the input parameter *method_outliers*. *thres_outl* is a threshold variable that is passed to the function to define outlier measurements. When *method_outliers* is set to “log.FC.distance” outliers are detected as the values in concentrations that are *thres_outl* times above or below the zero median reference value. When the method is “mad”, concentration values that deviate at least ± *thres_outl* median absolute deviations are considered outliers. When the method is “IQR” the upper and lower limits for outlier concentrations correspond respectively to (Q1—*thres_outl* x IQR) and (Q3 + *thres_outl* x IQR), where Q1 and Q3 are the first and third quartile values and IQR is the interquartile range.
  After outlier concentration values are detected, all samples containing at least one outlier value in their ion profile are removed from the data set. The user can also set the parameter *method_outliers* to “none” in order to skip the outlier analysis.
4. *Profile Standardisation* the normalised and filtered concentration values are then standardised. Two options are available through the input parameter *stand_method*. If it is equal to “std” the concentration values of each ion *m* are scaled by the standard deviation of all values, if equal to “mad” then the values are scaled by the median absolute deviation, which is a robust estimator of the std when the overall number of measurements is not very large. The user can also pass to the function a vector *stdev* of length *M* containing user-defined scaling values and set *stand_method* to “custom” for a custom standardisation.
  After standardisation the z-score profiles are aggregated at the line level by computing for each line the median value of the *m*-th element from the z-score profiles associated to the samples of that line.
5. *Profile Symbolisation* a value *thres_symb,* corresponding to a threshold in unit of standard deviation, is passed to the function and cell line symbolic profiles are extracted from their corresponding z-score profiles. The *m*-th element of the symbolic profile will be equal to + -1 if the corresponding element in the z-score profile is, respectively, above *thres_symb* or below -*thres_symb*, or otherwise it will equal 0.

The *PreProcessing* function provides the user with the following outputs:

- *stats.raw.data* a table containing the statistic of the unprocessed elemental concentrations measured.
- *stats.batches* a table containing the statistic of the batch corrected elemental concentrations across batches.
- *stats.outliers* a table containing the statistic of measurements detected as outlier.
- *stats.std* a table containing the scaling values used to standardise the elements.
- *data.long* a table containing the raw data of elemental concentrations.
- *data.line.logFC* a table containing batch corrected profiles (line aggregated).
- *data.line.zscores* a table containing z-score profiles (line aggregated).
- *data.line.symb* a table containing symbolic profiles (line aggregated).
- *plot.overview* a plot overviewing all values of elemental concentrations after batch correction and outlier detection.
- *plot.hist* a plot containing histograms describing the distribution of z-scores for each element.
- *plot.change.stat* a histogram plot describing the statistic of number of changed elements per cell line.
- *plot.change.dir* a histogram plot describing the statistic of increase and decrease of elemental abundances across cell lines.
- *plot.medians* a line plot of batch median values of element log-transformed concentrations.
- *plot.CV* a line plot of absolute coefficients of variation of the element log-transformed concentrations across batches.

### Analysis of ions

The *IonAnalysis* function is designed to perform an exploratory analysis of the elemental variability and of the correlations between the different measured ions using multivariate statistical methods including PCA and relevance networks inference (Butte & Kohane, 1999; Butte et al., 2000; Liang et al., 1998; Werhli et al., 2006). The function takes as input a data frame of z-score profiles of the type *data.line.zscores* returned by the *PreProcessing* function, a parameter *thres_ion_corr* representing a correlation threshold (default value set to 0.15), and a parameter *method_ion_corr* that specifies a measure to compute correlation coefficients compatible with the *cor* function (default method “pearson”) from the R package *stats (Team, *2019*)*. The function outputs the following results to the user:

- *data.pca.loads* a table containing the loadings of each element along the first two Principal Components.
- *plot.pca* an overview plot of the PCA analysis of the z-score profiles.
- *plot.corr* a correlation plot showing the pattern of element-element correlations extracted from the z-score profiles.
- *plot.net* a plot showing the relevance network between the elements given the input correlation threshold: nodes represent elements and the width of the link between two elements is proportional to their correlation coefficient.
- *plot.heat* a heatmap showing the clustering of rows and columns of the input z-score profile matrix using the R *hclust*(*stats*) method “ward.D” and the Euclidean distance, which has been shown to be a powerful nonlinear combination (Szekely & Rizzo, 2005).

### Clustering and network analysis of profiles

The *IonAnalysis* function focuses on the relations between the ions/features of the pre-processed data (columns of the data frame of z-score profiles). The *ProfileClustering* and the *GeneticNetwork* functions deal with the analysis of the z-score profiles to study the relations between the cell lines/observations of the data set.

The *ProfileClustering* function is designed to cluster lines based on the similarity of their symbolic profiles according to their Hamming distance. It takes as input a data frame of the type *data.line.symb* returned by the *PreProcessing* function, and uses three additional parameters: *min_clust_size*, an integer which corresponds to the minimal size in terms of number of lines that defines a cluster to be of interest (default equal to 10); *h_tree*, an integer which corresponds to the Hamming distance that defines the clusters (the default algorithm uses *h_tree* = 0 and groups together lines with identical symbolic profiles, meaning that their Hamming distance is zero); and *filtering_zero_string*, a logical parameter that removes from the input dataset the lines with all zeros in their symbolic profile, that can be interpreted as a cluster with no-phenotype of a phenotype consistent with the control lines.

*ProfileClustering* gives as output:

- *clusters.vector* a table containing the cluster id of each line.
- *tab.clusters* a table reporting the cluster size of each cluster (number of genes).
- *tab.clusters.subset* a table reporting the cluster size of each cluster (number of genes) only for clusters of a selected size (size greater than *min_clust_size*).

The *GeneticNetwork* function is designed (i) to extract a relevance network between the lines based on their profile similarity and (ii) to perform a graph analysis which includes community detection (Bianconi et al., 2014; Fortunato, 2010) and betweenness analysis (Latora et al., 2017), and (iii), to provide the means for network visualisation. The function is based on the methodology described in (Iacovacci et al., 2020). It takes as input a data frame of the type *data.line.zscores* and the following additional parameters: *method_corr* specifies a similarity/correlation measure to extract the relevance network; supported options include “pearson”, “spearman”, “kendall” from the *cor*(*stats*) R function, “cosine” from the *cosine*(*lsa*) R function (Wild, 2007), and “mahal_cosine” (Mahalanobis cosine) and “hybrid_mahal_cosine” (hybrid Mahalanobis cosine) (Iacovacci et al., 2020; Patil & Deore, 2014). The parameter *thres_corr* corresponds to the correlation threshold that define relevant similarities (the default value is set to 0.7). The parameter *network_modules*, can be set to “louvain”, in which case network modules are defined using the Louvain algorithm for community detection (Blondel et al., 2008), or “input”, in which case the network modules are passed as input to a *cluster_vector* object of the type *clusters.vector* returned by *ProfileClustering* and in addition cluster name labels can be passed as an object *cluster_label_vector*. The parameter *n_labels* is an integer proportional to how many nodes will be labelled in one of the output plots and is set to 3 as the default. R network packages used in the function include *igraph* (Csardi, 2013) and *network* (Butts, 2008).

*GeneNetwork* produces as output:

- *network* an edge list describing the network between the lines.
- *network.modules* a table containing network modules id’s of nodes.
- *stats.impact_betweenness* a table reporting the values of network betweenness and the impact of each line in the network. The impact is defined as the L_2_ norm of the z-score profile and it is an indicator of the overall deviation in elemental abundance.
- *plot.network* produces a plot showing the relevance network between the lines given the input correlation threshold.
- *plot.impact_betweenness* produces a scatter plot of network betweenness versus impact for each line.

### Enrichment analysis

In the case of dataset analysis where lines can be associated with genes (for example single gene knock-outs mutants) the user can perform a gene set enrichment analysis for KEGG metabolic pathways or GO Ontology terms by taking as gene sets the clusters obtained from the *ProfileClustering* or the network modules from the *GeneNetwork* function. The function is available for three different organisms, namely yeast, mouse and human.

The functions *KeggEnricher* and *GOEnricher* take as input a vector *cluster_vector* of the type *clusters.vector* or *network.modules*; a parameter *pval* specifying the p-value for the enrichment significance threshold; a character vector *gene_uni* specifying the gene universe list to be used (the default is NULL, in which case lines in *cluster_vector* also represent the gene universe); and a parameter *annot_pkg* which specifies the database to be used (for yeast *S.Cerevisiae* genes it has to be set to “org.Sc.sgd.db”, for mouse to “org.Mm.eg.db”, and for human to “org.Hs.eg.db”).

Additionally, the function *GOEnricher* takes as input a parameter *ont* to indicate which types of ontology should be used: “BP” for biological processes, “MF” for molecular functions, and “CC” for cellular components.

The genes in *cluster_vector* and *gene_uni* must be listed as ORF or ENTREZ IDS for yeast or ENTREZ IDS for human and mouse.

*KeggEnricher* and *GOEnricher* give as the output:

- *enrichment.summary* a table summarising the results from the enrichment analysis.
- *enrichment.full.results* a table containing cluster-specific details of the enrichment analysis.

## Results and discussion

### Case study 1: the genetic network of the *S. Cerevisiae* ionome

To illustrate the *IonFlow* pipeline we have used the iHUB Yeast Ionome data set (Yu et al., 2012), a large collection of population-average intracellular concentrations of 14 different elements (Ca, Cd, Co, Cu, Fe, K, Mg, Mn, Mo, Na, Ni, P, S, and Zn) quantified using ICP-MS in a collection of 4945 *S. cerevisiae* haploid mutant cell lines, where in each cell line a single non-essential ORF (open reading frame) was deleted. ICP-MS data were normalised by optical density (OD). Experimental details of the dataset are described in (Danku et al., 2009). Most of the cell lines (4207) were measured in 4 replicates, 684 lines in 8 replicates, 48 lines in 12 replicates, and 2 lines in 16 replicates giving a total of 26,976 samples screened in 305 different plates. Also, 4 control lines were present on the plates, generally in replicates of 4, namely BY4741, YDL227C, YLR396C, YPR065W.

ICP-MS raw data and OD corrected data can be downloaded from the iHUB (https://www.ionomicshub.org/yeast/beta/DataSearch.action). A table with the OD corrected data used in this study is available at https://github.com/wanchanglin/ionflow.

**Fig. 2:**
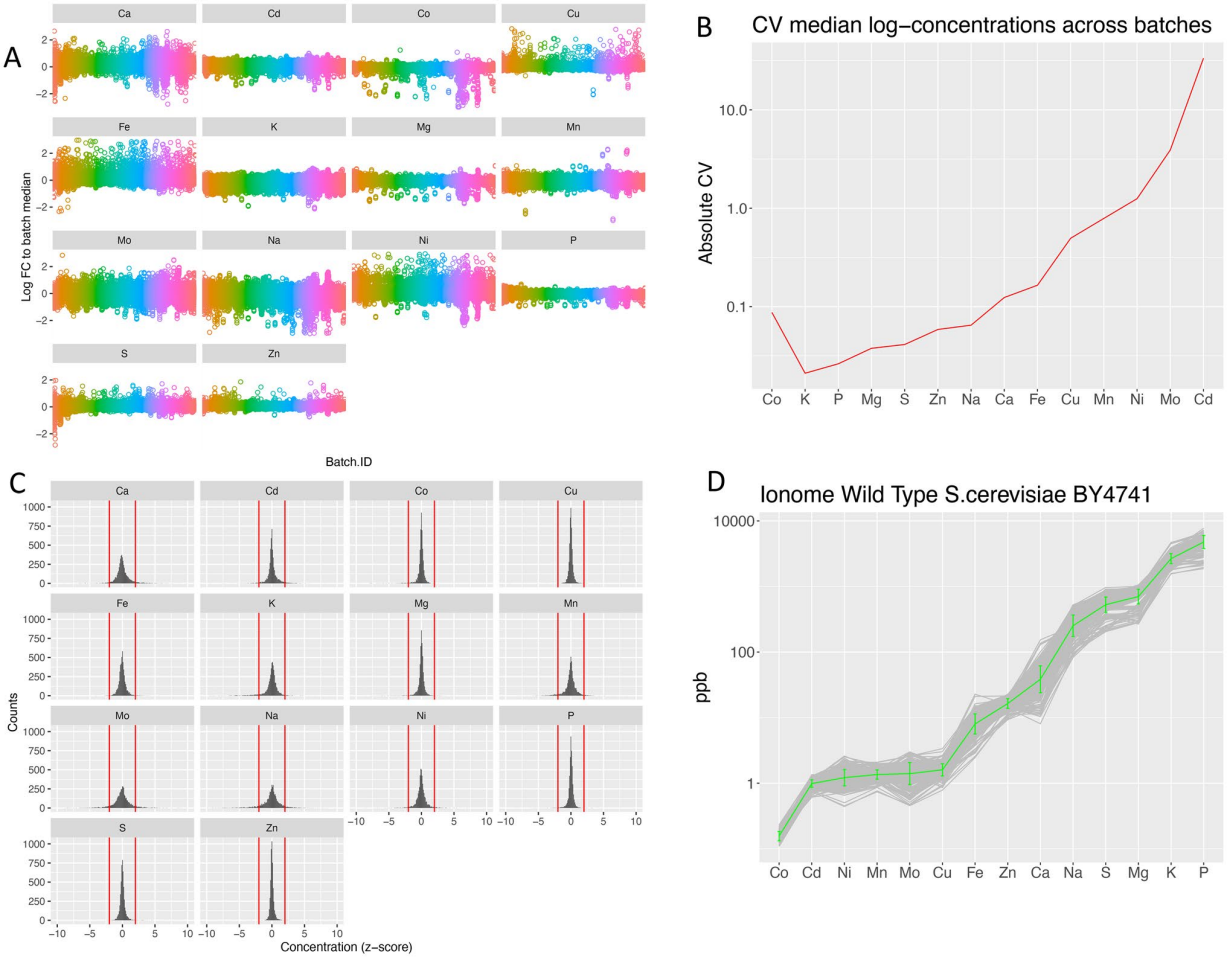
IonFlow diagnostic analysis of the yeast *S. Cerevisiae* ionome data set from the *PreProcessing* function. **A** The log-transformed normalised element concentrations are visualised across batches after outlier detection and removal. Different colours indicate different measurement plates (batches). **B** Absolute coefficient of variation of the elements across batches. **C** The distributions of the element z-score concentration values across the yeast mutants after standardisation together with the threshold (red vertical lines) set for the symbolisation. **D** The *S. Cerevisiae* BY4741 wild type ionome is shown, estimated by computing the mean value (green line) of the within-batch median concentrations (grey lines) (Color figure online)

In Fig. 2 we show some diagnostic output plots from the *PreProcessing* function after the raw data are processed. A plate-based normalisation of ion concentrations was enforced using the median value of all available lines in each plate. Outliers were defined as concentrations values deviating more than 3 times from the median after normalisation and all lines containing outlier values in their profiles were removed. Figure 2A shows the log-transformed normalised values across the plates (batches) after outlier detection and removal, and Fig. 2B shows the absolute coefficient of variation of the ions across plates (batches). The normalised profiles were standardised using the standard deviation of the ions measured across all samples and Fig. 2C shows the z-score distributions of the ions in the lines after standardisation together with the threshold set for the symbolisation (± 2 standard deviations).

We also repeated the *PreProcessing* using only the samples of the control line BY4741, a wild type yeast strain, and from the output table we converted back the in-plate median log-transformed concentration to the original part per billion (ppb) scale. In Fig. 2D the *S.Cerevisiae* BY4741 wild type ionome is shown, that was estimated from the mean value (green line) of the within-plate median measured concentrations (grey lines). The plot reveals that the elements with higher across-median CV (Fig. 2B) are, in general, the ones that are present in lower absolute concentration, as expected from an experimental perspective.

It is of interest to compare Fig. 2D with a previously reported diploid *S.Cerevisia* ionome quantification (Cyert & Philpott, 2013; Eide et al., 2005) to gain insights into the genetic background and the external cellular environment and how they play a role in the internal elemental balance of the cell. Despite the level of potentially toxic elements such as cadmium and sodium being artificially increased in the yeast growth media for the data set under study (Danku et al., 2009) most of the elements agree in order of magnitude with the level reported in (Cyert & Philpott, 2013).

**Fig. 3:**
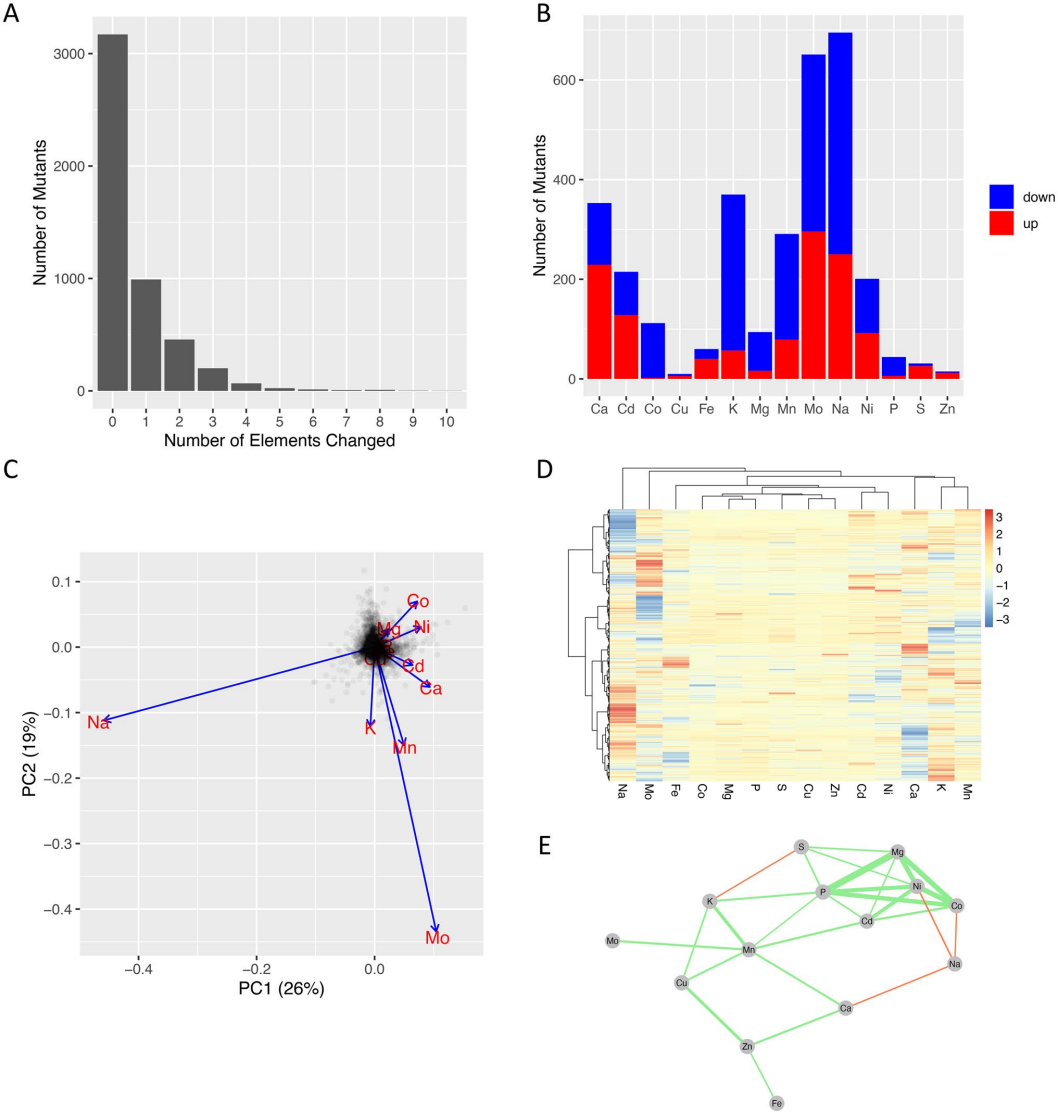
Elemental analysis of the yeast *S. Cerevisiae* ionome data set. **A** The statistic of the number of altered elements per yeast knockout mutant; 75% of mutants do not show any change at the level of their symbolic profile given a ± 2 z-scores threshold, and the probability of having a profile with *k* altered elements decreases exponentially with *k*. **B** The statistic of changes by element, together with the change of direction (increase or decrease in concentration with respect to the mean). **C** mutant elemental profiles are visualised in the plane of PC1 vs PC2; the projected eigenvector length (blue vectors) is proportional to the variance of the elements and the angle between the projected eigenvectors is proportional to the correlation of abundances across the mutants between the corresponding elements. **D** Hierarchical clustering of the z-score profiles. **E** Relevance network (relevance threshold 0.15) describing the element-element correlations: green/red links indicate positive/negative correlations respectively, and the width of the link is proportional to the absolute correlation value (Pearson’s correlation) (Color figure online)

We then proceeded to the analysis of the processed z-score profiles and symbolic profiles from the *PreProcessing* function. In Fig. 3A, B the *data.line.symb* table is used to produce a histogram plots that describe the statistics of changes in the yeast ionome for the selected symbolic threshold. Figure 3A reveals that most lines (3171, ~ 75%) do not show any change at the level of their symbolic profile, and that, for most of the remaining lines that show a phenotype, the probability of having a profile with *k* altered ions decreases exponentially with *k*. Figure 3B illustrates the changes by element, together with the change direction, and it is interesting to note that copper, zinc, iron and sulphur, that have an essential role in the cell in shaping protein structure/function and acting as enzyme cofactors (Cyert & Philpott, 2013), are the elements that are less likely to be altered and that also show a preferential increase in the directionality of the change, suggesting that robust mechanisms must exist in *S.cerevisiae* to control the homeostasis of these elements and that their decrease is likely to produce unviable mutants.

This elemental analysis was extended via the *IonAnalysis* function (in Fig. 3C–E the output plots are shown). The PCA plot (Fig. 3C) shows, as expected, that the projected eigenvector length (blue vectors) is proportional to the variance of that element in the data (Fig. 3B) and that the smaller the angle between the projected eigenvectors, the more the ions are likely to be clustered together when hierarchical clustering analysis is performed using their z-score profile across the lines (Fig. 3D). Figure 3E depicts the relevance network extracted from the element-element correlations using the default threshold and Pearson’s correlation coefficient (green links indicate positive correlations, while red links indicate negative correlations). Mg and P, that show a preferential change in direction towards a decrease in intracellular concentration, are the most correlated elements and are significantly clustered with Ni and Co, and Cd, which is consistent with results reported in (Eide et al., 2005). It is interesting to note that the observed correlation is likely to reflect ATP consumption in activation of gene-deletion compensatory mechanisms, given that most of the Mg^2+^ intracellular ions are bound to ATP and ribosomes (Milo & Phillips, 2015). Na and K, that are mostly found within the cell as monovalent ions Na^+^ and K^+^, are weakly correlated to other elements, and the fact that sodium appears to be mostly anticorrelated with other elements might reflect the difficulty in its quantification due to various contamination sources (Milo & Phillips, 2015).

The symbolic profiles were then clustered using the *ProfileClustering* function with default parameter choice and the resulting cluster vector was given as input as the modules vector to the *GeneticNetwork* function together with the z-score elemental profiles in order to perform a network analysis of the profile correlations between the mutant lines. For this analysis the cosine similarity was used as the correlation measure and a relevance correlation threshold of 0.7 was chosen.

**Fig. 4:**
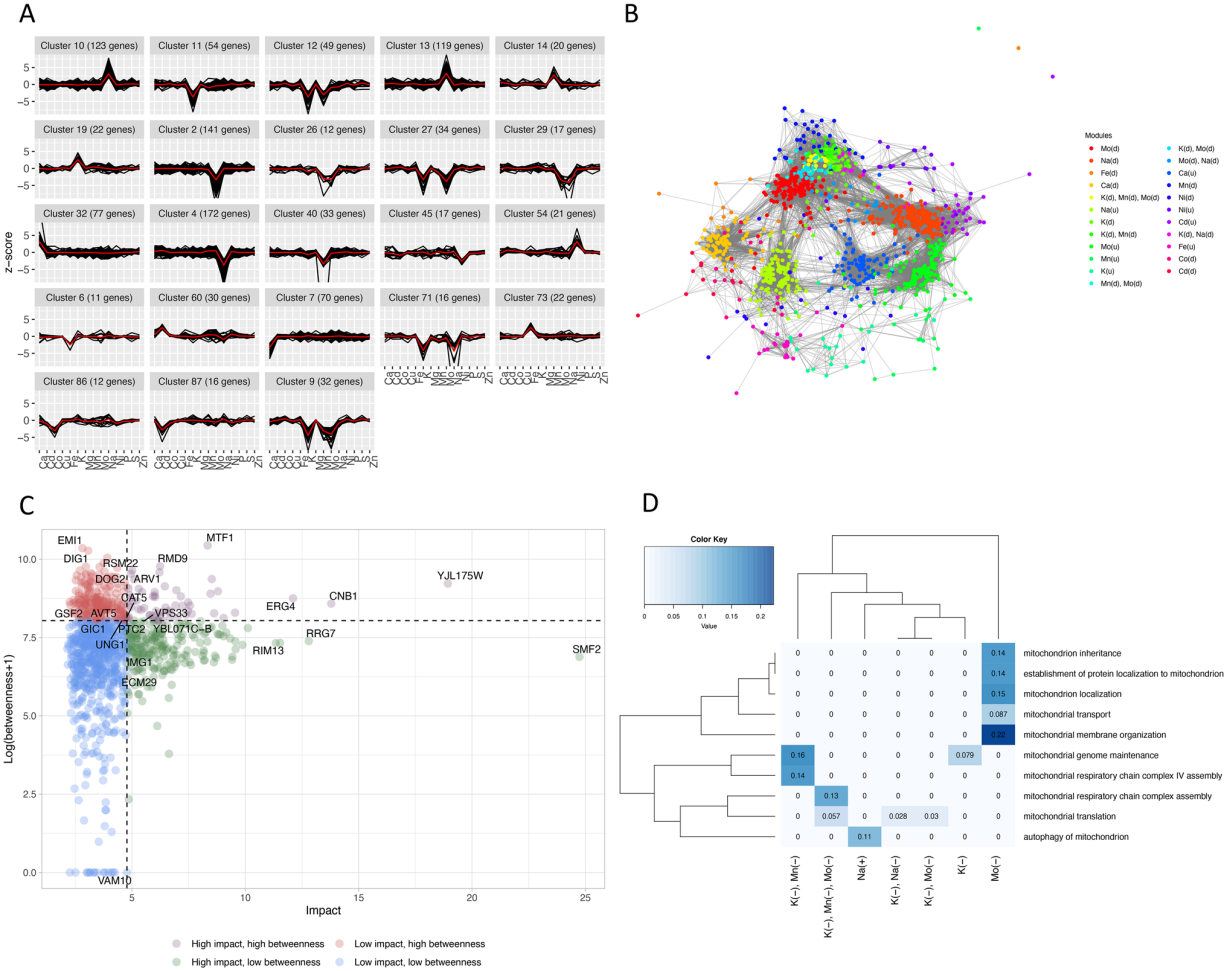
Mutant elemental profile analysis of the yeast *S. Cerevisiae* ionome data set. **A** The z-score profiles (grey lines) of the mutants revealing the most common phenotypes are shown together with the average within-cluster profile (red lines); the clustering, based on the similarity between the mutant’s symbolic profiles, was performed using the *ProfileClustering* function. **B** The genetic relevance network inferred from elemental profile correlations between the knockout mutants found in the most common phenotypes is shown (cosine similarity, relevance threshold 0.7); the nodes represent genes, and the edges represent a genetic association (correlated elemental profile in response to deletion); the network modules correspond to the phenotype clusters in A, with labels describing the cluster features in terms of altered elemental levels (‘u’ indicates up level, ‘d’ indicates down level). **C** Impact versus network betweenness analysis of the network nodes; the impact is defined as the L_2_ norm of the z-score profile. **D** Gene clusters enrichment analysis performed with the *GOEnricher* function; the heatmap shows the percentage of universe genes annotated for all enriched mitochondrial terms in function of the cluster phenotypic characteristics (Color figure online)

Figure 4A shows the z-score profiles of the lines clustered according to *ProfileClustering* for all clusters with a minimum size of 10 lines corresponding to the most common ionome phenotypes (average within-cluster profiles are shown in red). Figure 4B shows a visualisation of the genetic network inferred from elemental profile correlations between the knock-out mutants found in the most common phenotypes. Nodes represent genes, edges represent a genetic association (deletion produces a similar phenotype), and the network modules correspond to the clusters in Fig. 4A, with labels describing the cluster features in terms of altered elemental levels (‘u’ indicates up, ‘d’ indicates down). Figure 4C reports the plot of impact versus betweenness for all nodes in the network. The impact is defined as the L_2_ norm of the z-score profile and it is a measure of the overall alteration of a profile, while the betweenness quantifies the role of a node in bridging between modules. Nodes are categorised and coloured according to high/low impact and high/low betweenness. These in turn are determined by including in the high group the 25% highest values of each descriptor. The 3 nodes with highest impact and the 3 nodes with highest betweenness are also labelled within each category. SMF2, a very important manganese transporter, has the highest impact, with a z-score for Mn of approximately − 25 (25 standard deviations below the median value). MTF1, a mitochondrial gene from cluster 10, which shows reductions of K and of Na levels, is the gene with highest betweenness.

Finally, the *GOEnricher* function was used to perform a biological process enrichment analysis of the common clusters. Results revealed that several of these clusters were enriched by mitochondrial related processes suggesting that mitochondrial dysfunction is associated with disruption of ion homeostasis.

The heatmap in Fig. 4D shows the percentage of genes annotated for given mitochondrial terms that are found inside each cluster enriched for that term. These results imply that the genome partition obtained from the ionome data is biologically informative and that mitochondrial enriched clusters are in general associated with reduction of specific elements; namely potassium, sodium, manganese and molybdenum.

### Case study 2: the HeLa ionome

To provide a second illustrative example we processed another ionome data set that describes concentrations of trace elements in human HeLa cells obtained through a genome-wide high-throughput siRNA/ionomics screen (Malinouski et al., 2014). Data were not pre-processed, instead the z-score profiles provided by the authors of the study were used as additional benchmark data for the elemental analysis and for the genetic network analysis. The z-score data contains normalised and standardised concentration measurements for 18 elements (As, B, Ca, Cd, Co, Cu, Fe, K, Li, Mg, Mn, Mo, Na, Ni, P, S, Se, and Zn) profiled for 775 mutant lines of HeLa cells, each having a single different gene silenced.

**Fig. 5:**
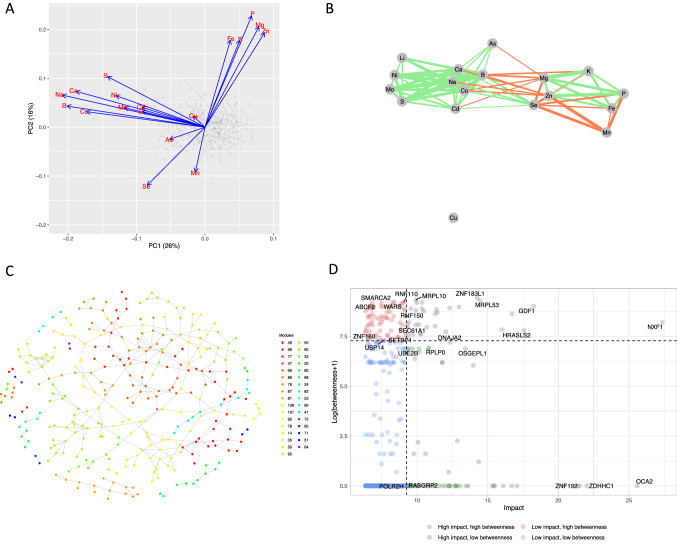
IonFlow analysis of the human ionome data set from HeLa cells. **A** PCA of the elemental profiles of the knockdown mutants. **B** The relevance element-element correlation network is shown (Pearson’s correlation, relevance threshold 0.15). **C** The genetic relevance network between selected high-impact elemental profiles of knockdown mutants is depicted (Mahalanobis cosine similarity, relevance threshold 0.6); nodes are coloured according to network modules found via network community detection. **D** Impact-betweenness analysis of the knockdown mutants

In Fig. 5A, B results from the ion analysis are shown. The profiles projected onto the first two principal components, along with the ion loadings are shown in Fig. 5A while the relevant ion-ion correlation network is displayed in Fig. 5B. While some relations between the ions such as correlated levels of Mg and P can still be identified in this ionome, the overall interpretation is difficult, probably due to the cancerous nature and rapid proliferation of the HeLa cells (Pavlova & Thompson, 2016). The genetic network analysis is illustrated in Fig. 5C, D. All profiles with an impact below the median impact value were filtered out (387 lines). The genetic network (Fig. 5C) between the selected lines was extracted using a relevance threshold of 0.6 and the Mahalanobis cosine as a correlation measure, which is more effective when profiles are characterised by an extended, dense pattern of element-element correlations (Fig. 5B) such as in this case. Nodes are coloured according to network modules assessed by the Louvain community detection algorithm. Isolated nodes are not visualised in the plot. Table 1 reports the results of the enrichment analysis for KEGG metabolic pathways obtained with the *KEGGEnricher* function for the network modules.

**Table 1:** results of the KEGG pathway enrichment analysis on the network modules of the genetic network extracted from the HeLa cell ionome

Cluster	KEGGID	P value	Count	Size	Term
69	3013	0.06249663	3	20	RNA transport
81	52	0.0016808	3	6	Galactose metabolism
81	4910	0.00735846	4	18	Insulin signaling pathway
81	4114	0.01940962	3	13	Oocyte meiosis
106	4114	0.01387635	3	13	Oocyte meiosis
101	10	0.00088208	4	12	Glycolysis/gluconeogenesis
101	4270	0.0084409	3	11	Vascular smooth muscle contraction
78	3050	0.0674506	4	21	Proteasome
95	4666	0.0464702	3	14	Fc gamma R-mediated phagocytosis
95	4810	0.07688909	3	17	Regulation of actin cytoskeleton
55	5016	0.02274157	4	20	Huntington's disease
55	4141	0.04970889	3	15	Protein processing in endoplasmic reticulum
55	240	0.07943879	3	18	Pyrimidine metabolism
60	4270	0.02379386	3	11	Vascular smooth muscle contraction

In Fig. 5D the impact-betweenness analysis of the knockdown mutants is plotted. Among the high impact and high betweenness genes is NFX1, that plays a role in the export of mRNA of the HSP70 family (whose members are known to become strongly upregulated by heavy metals such as arsenic, cadmium and copper) and MRPL53, a component of the mitochondrial large ribosomal subunit.

## Conclusion

We presented IonFlow, a tool that makes the analysis of ionomics data accessible to Galaxy users and that allows them to quickly explore, visualise, and interpret their data via multivariate approaches used in the field of ionomics including PCA, correlation analysis, network inference and enrichment analysis. IonFlow also incorporates recently developed methods for ionome data sets, such as ion-profile-specific similarity measures (Iacovacci et al., 2020) that optionally, can be used by the user. Each function of IonFlow was described in detail and the pipeline was tested on two large benchmark ionome datasets, the ionome of haploid *S.Cervisieae* and the ionome of HeLa human cells, to illustrate its applicability and its outputs within two concrete case studies.

We showed that IonFlow is very versatile and it can be used to process raw data as well as directly process normalised and standardised data for advanced analysis such as genetic network extraction and study of element-element correlations. For these reasons IonFlow is of interest for researchers dealing with ionomics experiments beyond those performed with ICP–MS technology and its applicability potentially extends to the analysis of metabolomics data.

## Supplementary Information

Below is the link to the electronic supplementary material.

Supplementary file1 (PDF 1754 kb)

## Declarations

### Data availability

Data and R codes are freely accessible at https://github.com/wanchanglin/ionflow.
