# Supplementary material for: IonFlow: a galaxy tool for the analysis of ionomics data sets
Source: Metabolomics. 2021 Sep 25;17(10):91. doi: 10.1007/s11306-021-01841-z (PMC8464566; doi:10.1007/s11306-021-01841-z)
Supplement: Supplementary file 1 — Supplementary file1 (PDF 1754 kb) [file 11306_2021_1841_MOESM1_ESM.pdf]

Supplementary Information

IonFlow: a Galaxy Tool for the Analysis of Ionomics Data Sets

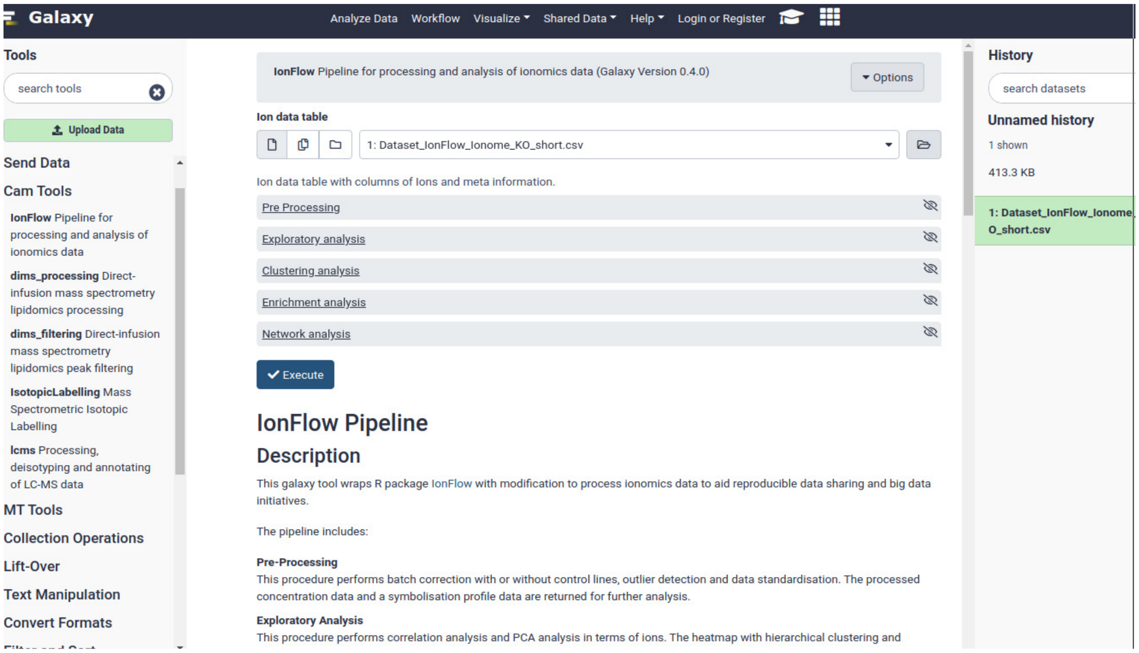

Figure S1. Galaxy tool IonFlow interface. The left pane shows the Galaxy tools including Upload Data and IonFlow. The centre pane indicates IonFlow five sections: Pre Processing, Exploratory Analysis, Clustering Analysis, Enrichment Analysis and Network Analysis. The right pane lists the data loading.

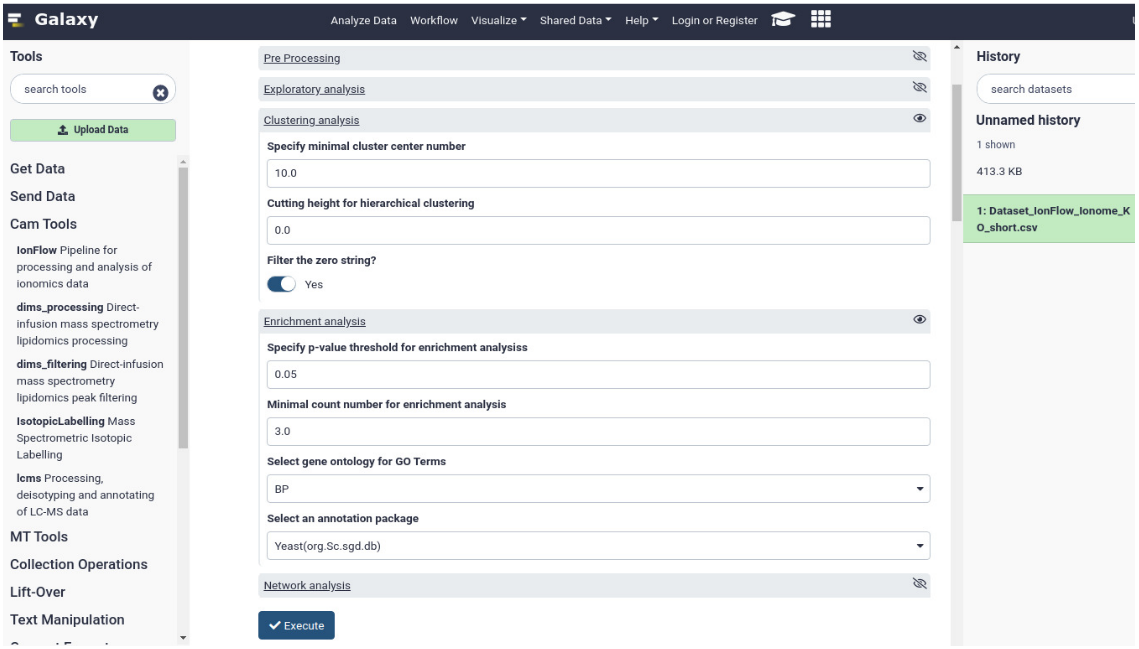

Figure S2: Galaxy tool IonFlow interface. The centre pane shows two collapsed sections: Clustering analysis and Enrichment, with parameters setting. To collapse a section, click the section title.

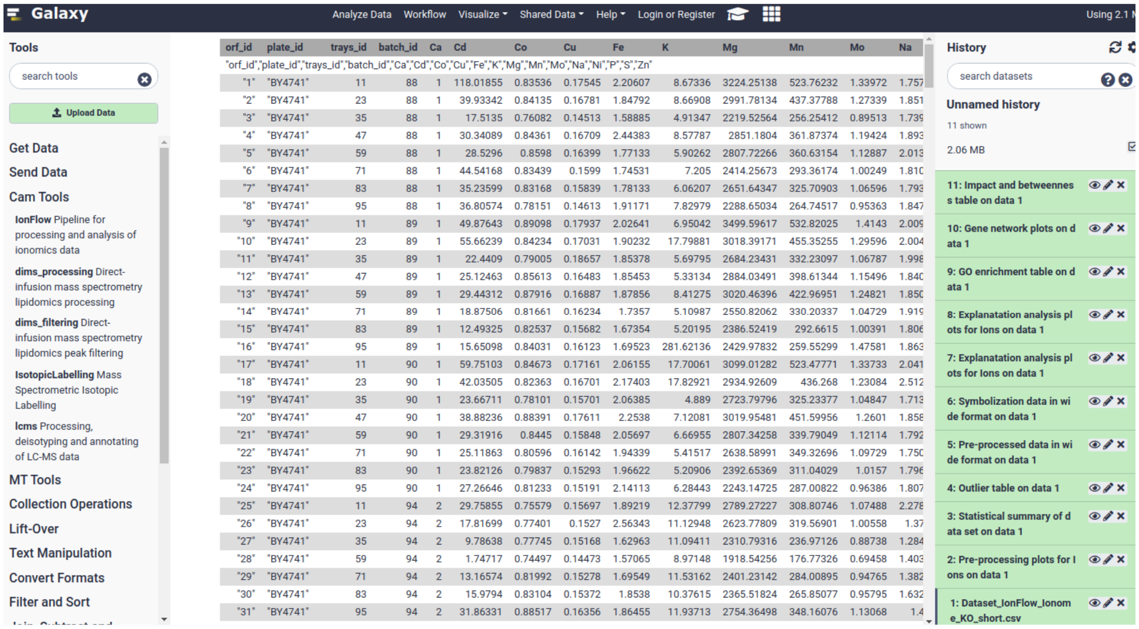

Figure S3: Galaxy IonFlow tool data loading. This figures shows the contents of an ionomics data. Currently supported data format is comma-separated values (CSV).

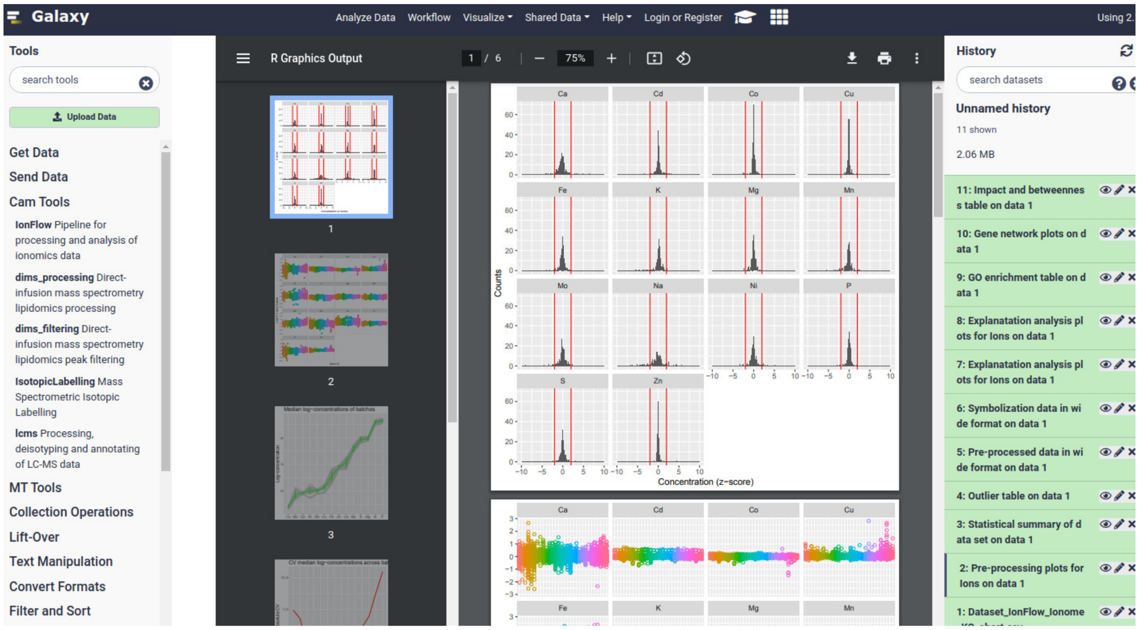

Figure S4: Galaxy tool IonFlow result pane. This figure shows the results of Pre-Processing.

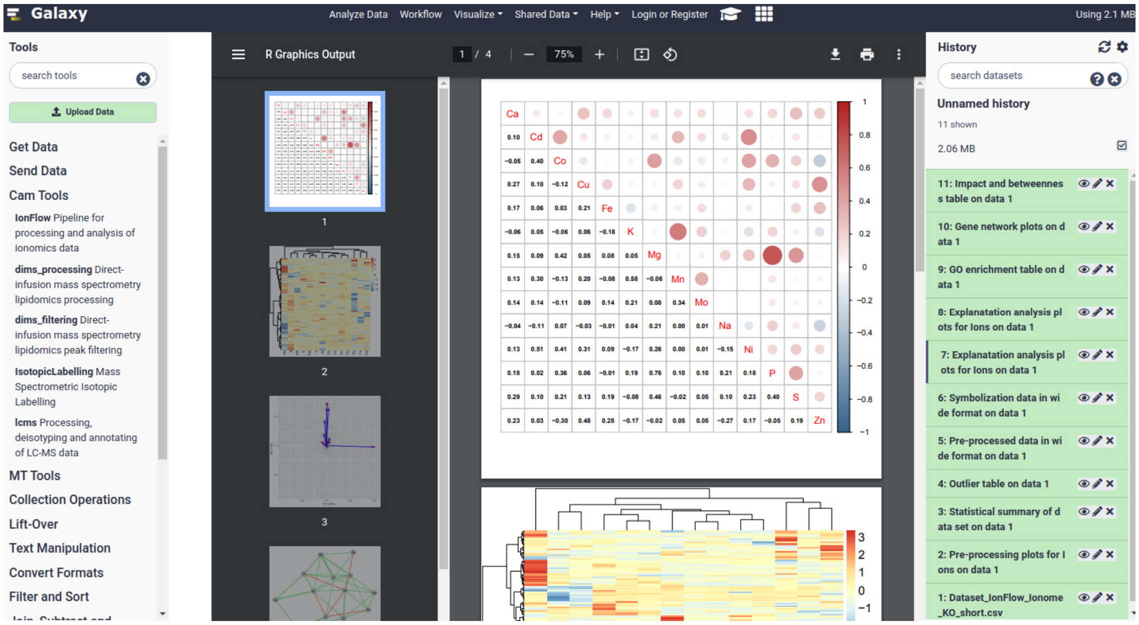

Figure S5: Galaxy tool IonFlow result pane. This figure shows the results of Exploratory Analysis.

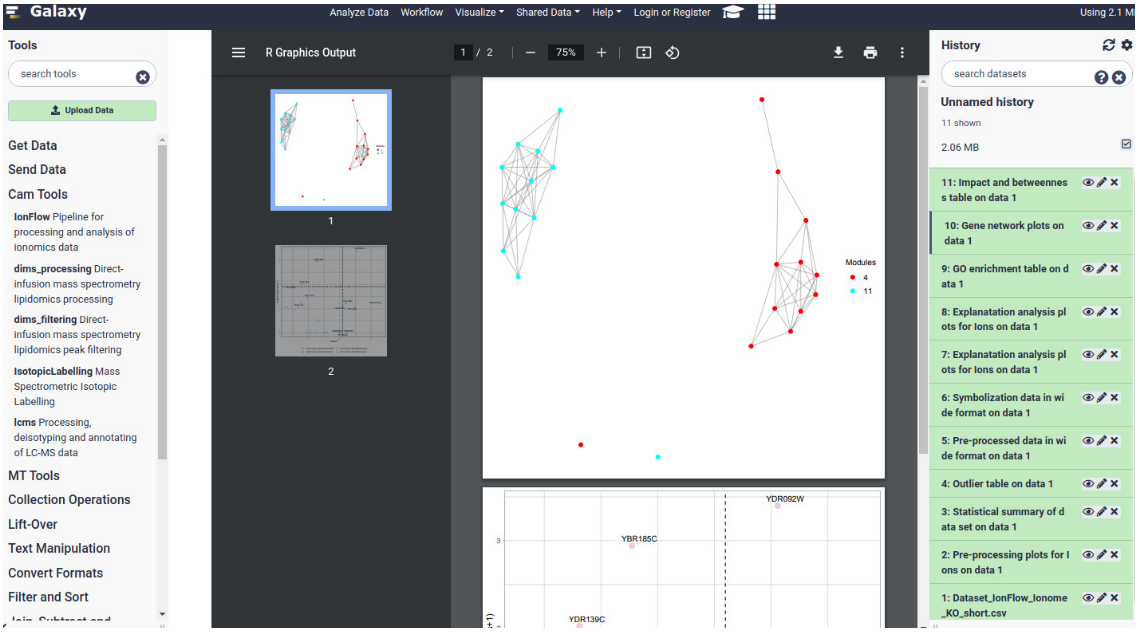

Figure S6: Galaxy tool IonFlow result pane. This figure shows the results of Network Analysis.
